# Association of Trace Element Levels with Outcomes in Critically Ill COVID-19 Patients

**DOI:** 10.3390/nu15153308

**Published:** 2023-07-26

**Authors:** Hannah Wozniak, Christophe Le Terrier, Steve Primmaz, Noémie Suh, Sébastien Lenglet, Aurélien Thomas, Nicolas Vuilleumier, Sabrina Pagano, Aude de Watteville, Silvia Stringhini, Idris Guessous, Hervé Quintard, Claudia Paula Heidegger, Jérôme Pugin

**Affiliations:** 1 Division of Intensive Care, Geneva University Hospitals, the Faculty of Medicine, University of Geneva, 1205 Geneva, Switzerlandaude.dewatteville@hcuge.ch (A.d.W.); herve.quintard@hcuge.ch (H.Q.); claudia.heidegger@hcuge.ch (C.P.H.);; 2 Forensic Toxicology and Chemistry Unit, CURML, Lausanne University Hospital, Geneva University Hospitals, 1205 Geneva, Switzerlandaurelien.thomas@chuv.ch (A.T.); 3 Faculty Unit of Toxicology, CURML, Faculty of Biology and Medicine, University of Lausanne, 1015 Lausanne, Switzerland; 4 Division of Laboratory Medicine, Diagnostics Department, Geneva University Hospitals, Faculty of Medicine, University of Geneva, 1205 Geneva, Switzerland; 5 Unit of Population Epidemiology, Division of Primary Care Medicine, Geneva University Hospitals, 1205 Geneva, Switzerland; 6 Department of Community Medicine, Primary Care and Emergency Medicine, Geneva University Hospital, 1205 Geneva, Switzerland

**Keywords:** COVID-19, copper, selenium, zinc, intensive care unit, trace elements, mortality

## Abstract

The primary objective of this study was to compare the plasma levels of copper, selenium, and zinc between critically ill COVID-19 patients and less severe COVID-19 patients. The secondary objective was to investigate the association of these trace element levels with adverse outcomes, including the duration of mechanical ventilation, occurrence of septic shock, and mortality in critically ill COVID-19 patients. All COVID-19 patients admitted to the ICU of the Geneva University Hospitals between 9 March 2020 and 19 May 2020 were included in the study. Plasma levels of copper, selenium and zinc were measured on admission to the ICU and compared with levels measured in COVID-19 patients hospitalized on the ward and in non-hospitalized COVID-19 patients. To analyze the association of trace elements with clinical outcomes, multivariate linear and logistic regressions were performed. Patients in the ICU had significantly lower levels of selenium and zinc and higher levels of copper compared to COVID-19 patients hospitalized on the ward and in non-hospitalized COVID-19 patients. In ICU patients, lower zinc levels tended to be associated with more septic shock and increased mortality compared to those with higher zinc levels (*p* = 0.07 for both). Having lower copper or selenium levels was associated with a longer time under mechanical ventilation (*p* = 0.01 and 0.04, respectively). These associations remained significant in multivariate analyses (*p* = 0.03 for copper and *p* = 0.04 for selenium). These data support the need for interventional studies to assess the potential benefit of zinc, copper and selenium supplementation in severe COVID-19 patients.

## 1. Introduction

The symptoms of coronavirus disease 2019 (COVID-19) include a range of mild to severe respiratory symptoms that may lead to hospitalization, with the most severe patients requiring admission to the intensive care unit (ICU) for advanced respiratory support [1]. COVID-19 generates a systemic inflammatory response that can lead to organ dysfunction including the acute respiratory disease syndrome (ARDS). High levels of inflammation are associated with an increased mortality rate [2,3], and only a few immunomodulatory therapies seem to reduce mortality in these patients [4,5].

Several risk factors for developing severe COVID-19 such as age, obesity, diabetes and cardiovascular disease have already been identified [6]. Trace elements have also been studied in this context, and low zinc and selenium levels have been identified as potential prognostic factors in both hospitalized COVID-19 patients [7,8], and in critically ill COVID-19 patients [9,10]. In contrast, data on copper remain scarce. Furthermore, the variations in trace element levels among COVID-19 patients with different degrees of severity have not been described. The trace elements zinc, copper and selenium are essential dietary micronutrients that participate in protein synthesis and cell signaling. They also inhibit viral replication, play important roles in immune homeostasis, in particular, in the control of excessive inflammatory responses, and carry antioxidants effects [11]. Trace elements have also a significant role in the synthesis of viruses. For instance, zinc has been identified as an inhibitor of viral polymerases, including coronavirus [12,13,14,15,16,17]. Supplementation with these trace elements has been proposed as an adjuvant therapy in COVID-19 with conflicting results [18,19].

The purpose of this study was to compare plasma levels of copper, zinc and selenium in a COVID-19 cohort with varying severity of illness, ranging from non-hospitalized to critically ill patients, and to determine a possible association between these plasma levels and time under mechanical ventilation (MV), septic shock and death.

## 2. Materials and Methods

### 2.1. Study Design and Participants

This single-center retrospective observational study was conducted in the ICU of Geneva University Hospitals (Geneva, Switzerland), between 9 March 2020 and 19 May 2020. All adult patients admitted to the ICU with acute respiratory failure due to SARS-CoV-2 infection were included. SARS-CoV-2 infection was defined by a positive reverse transcriptase-polymerase chain reaction test on a nasopharyngeal swab and/or in bronchoalveolar lavage (BAL) fluid. Severe COVID-19 patients were defined by patients requiring an admission to the ICU for mechanical ventilation or high flow nasal oxygenation with an inspired fraction of oxygen > 80% [20].

In order to compare trace elements levels among patients with different disease severity, the following three cohorts were constituted: (1) critically ill COVID-19 patients hospitalized in the ICU (*n* = 119), (2) severe COVID-19 patients hospitalized on the ward (*n* = 98), and (3) COVID-19 outpatients from the SEROCoV-POP cohort (*n* = 129) [21]. Patients included in the groups (2) and (3) were randomly selected among COVID-19 patients hospitalized on the ward during the same period and among seropositive participants of the SEROCoV-POP cohort, respectively. Patients included in those groups were matched using age, gender and BMI.

The Institutional ethics committee approved the study (Swiss BASEC number: 2020-00917). An informed consent was obtained either from the patient or the next of kin.

### 2.2. Data Collection

In the cohort of ICU patients, all demographic characteristic, severity scores and biological data were collected at the time of ICU admission. Therapies such as mechanical ventilation (MV), prone positioning and extracorporeal membrane oxygenation (ECMO), as well as complications such as septic shock, and thrombotic events were recorded during ICU stay. Septic shock was defined according to Sepsis-3 definitions [22]. Patients were followed until hospital discharge or death. Survival status was analyzed at day 28.

#### Determination of Trace Element Levels

Among groups of ward-hospitalized and outpatient patients, only trace elements were measured. Plasma levels of zinc, copper and selenium were measured on the day of ICU admission (group 1), on the day of hospital admission (group 2) and on the day of ambulatory visit (group 3). All plasma samples were analyzed in the same laboratory using the same analytic technique.

Trace element concentrations were measured in human plasma via inductively coupled plasma mass spectrometry (ICP-MS; 7800 Series; Agilent, Palo Alto, Santa Clara, CA, USA) as described previously [23,24] for elementary quantification of copper, selenium and zinc. Details on the measurement methods are provided in Supplementary Tables S1–S3. Briefly, 300 µL of plasma was diluted with 2.7 mL of HNO_3_ 0.1% solution containing 10 ng/mL Rhodium and 10 ng/mL Indium as internal standards. In addition, each analytical batch of study samples was processed with laboratory controls, including method blanks and standard reference materials to continuously monitor method performance. The normal range of trace elements in serum was defined as 10–23 µmol/L for zinc, 12.5–23.6 µmol/L for copper and 0.95–1.6 µmol/L for selenium, according to our hospital guidelines, based on data from the Swiss population.

### 2.3. Statistical Analysis

Trace element levels among the three severity groups were expressed as median and interquartile range (IQR). Comparison between trace elements levels among these groups was performed using a one-way analysis of variance (ANOVA), with the Bonferroni test for multiple comparisons.

Patients’ characteristics at the time of ICU admission were collected. Continuous variables were presented as median and IQR, and categorical variables were expressed as the number of patients (*n*) and percentage (%). Chi-square test were used to detect differences in categorical variables and Mann–Whitney *U* test in continuous variables.

To assess the association between lower levels of trace elements and the clinical outcomes of time under MV, septic shock and death, patients were separated into two groups according to the median level of each trace element. Patients above the median were classified as the normal trace element group, and those below the median were classified as the low trace element group. We performed descriptive analyses of patients’ characteristics and outcome, i.e., septic shock, length of MV and death, according to the levels of trace elements. Pearson’s correlation analysis was performed to assess the associations among copper, zinc and selenium levels.

A multivariable linear regression was performed to characterize the association between low trace elements levels at ICU admission and time under MV. A multivariable logistic regression was performed to characterize the association between low trace elements levels at ICU admission and septic shock during ICU stay and day 28 mortality. The following clinically relevant variables were included in the multivariable analysis: gender, SAPS II and the presence of any comorbidity. Results of the linear regression are expressed as linear coefficient (coeff.) with 95% confidence interval (CI 95%). Results of the logistic regression are expressed as odds ratio (OR) and 95% confidence interval (CI 95%). Two-tailed *p*-values ≤ 0.05 were considered statistically significant. All statistical analyses were conducted using STATA, version 16.1 (Stata Corp., College Station, TX, USA, 2007).

## 3. Results

### 3.1. Trace Element Levels According to Disease Severity

The COVID-19 study population was composed of 129 outpatients, 98 patients hospitalized on the ward and 118 ICU patients. Median levels of zinc were 19.2 (16.6–22.9) µmol/L, 13 (11.1–15.4) µmol/L and 8.2 (6.9–9.7) µmol/L for outpatient, ward-hospitalized and critically ill patients, respectively. Selenium levels were 1.5 (1.4–1.7) µmol/L, 1.1 (0.8–1.4) µmol/L and 0.8 (0.7–1) µmol/L and levels of copper were 15.6 (13.9–18.2) µmol/L, 17.5 (14.1–21.3) µmol/L and 18.3 (16.1–20.5) µmol/L in these groups.

**Figure 1:**
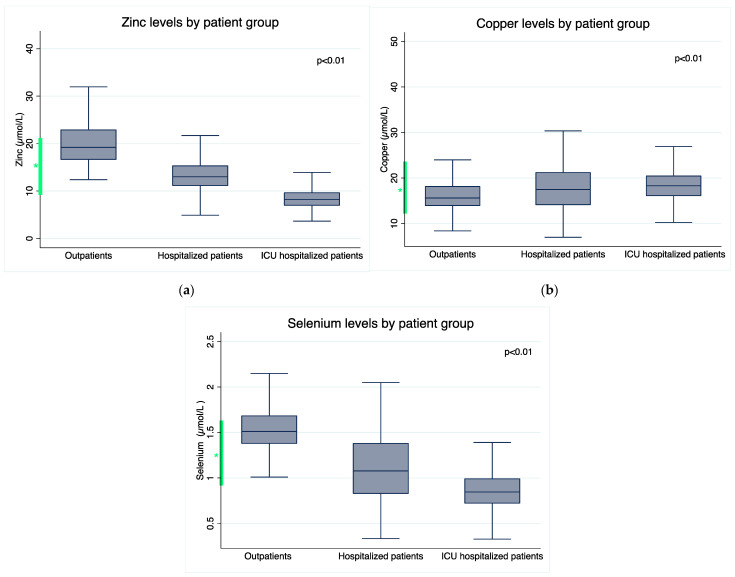
Trace element plasma levels according to COVID-19 severity. (**a**) Plasma zinc levels. Difference in trace elements plasma levels between groups (one-way ANOVA). Bonferroni post hoc test: outpatients > hospitalized patients > ICU hospitalized patients; (**b**) plasma copper levels. Difference in trace elements plasma levels between groups (one-way ANOVA). Bonferroni post hoc test: outpatients < hospitalized patients, outpatients < ICU hospitalized patients; (**c**) plasma selenium levels. Difference in trace elements plasma levels between groups (one-way ANOVA). Bonferroni post hoc test: outpatients > hospitalized patients > ICU hospitalized patients. * The green interval in the figures refers to the laboratory range references.

Differences in levels of each trace element in comparison with the normal range and between groups are presented in Figure 1. Zinc, selenium and copper levels varied significantly between the groups (*p* < 0.01).

### 3.2. Baseline Characteristics of ICU COVID-19 Patients

Baseline characteristics are described in Table 1. Among the ICU population, 91/118 (77.1%) were men. Median plasma levels of copper, zinc and selenium were 18.3 (16.2–20.5) µmol/L, 8.2 (6.9–9.7) µ/L and 0.8 (0.7–1) µmol/L, respectively.

### 3.3. Association between Plasma Levels of Trace Elements and Outcomes

Supplementary Table S4 shows the repartition of ICU patient according to their trace element level. Patients with lower selenium and zinc levels were older and presented with higher CRP levels on ICU admission. Patients with lower copper levels were more often male. Pearson’s correlation analysis revealed significant correlations between each measured micronutrient (*p* < 0.01 for all) (Supplementary Table S5).

The association between lower zinc, copper and selenium levels and outcomes is presented in Table 2. Patients with low zinc levels tended to present more septic shock and tended to have a higher mortality (*p* = 0.07 for both). Patients with low copper levels had longer time under MV (*p* < 0.01). Patients with low selenium tended to have longer time under MV and higher mortality (*p* = 0.09 and *p* = 0.07, respectively).

Results of the multivariate analysis are shown in Table 3. There was a non-significant association between low zinc level and septic shock (*p* = 0.06). Patients with low copper and selenium levels had a statistically significantly longer time under MV (*p* = 0.03 and *p* = 0.04, respectively).

## 4. Discussion

Herein, we show that COVID-19 patients presented different plasma trace elements levels depending on disease severity. In the ICU population, selenium and zinc plasma levels measured on ICU admission were lower, whereas copper levels were higher at the time of ICU admission, compared to COVID-19 patients on the ward or to COVID-19 outpatients. In critically ill patients, lower zinc levels on ICU admission tended to be associated with increased septic shock and mortality, and lower levels of copper and selenium tended to be associated with prolonged time under MV. The association between lower copper and selenium levels and time under MV remained significant in a multivariate model.

The lower levels of zinc, selenium and copper found in critically ill patients compared to COVID-19 patients hospitalized on the ward and COVID-19 outpatients, and its association with adverse clinical outcomes, are consistent with other studies from Switzerland [8], France [25], and Belgium [26]. Whereas previous studies evaluated the trace elements only on healthy people or on a small number of critically ill patients [7,26], our study is original in that it describes those levels in COVID-19 patient populations of various degrees of severity, and its association with clinical outcomes [7,27].

In contrast to zinc and selenium plasma levels, which were lower in severe patients, copper levels were higher in critically ill COVID-19 patients when compared with both outpatients and hospitalized patients. These results differ from those of a previous study that reported low copper levels at ICU admission for all causes and highlighted an association with mortality [28]. As copper is mostly bound to the acute phase protein ceruloplasmin elevated in plasma during inflammatory processes, it is likely that copper, in contrast to other trace elements, accumulates in the plasma with concentrations rising during inflammatory states, which could explain our findings [12].

An important finding of our work is the association between low trace element levels in the plasma and adverse clinical outcome in critically ill COVID-19 patients. Trace elements play an important role in the synthesis of viruses, zinc being for example an inhibitor of viral polymerases, including coronavirus [12,13,14,15,16,17]. Zinc is necessary for the function of over 200 metalloproteases, and plays a role in immunity, modulation of the inflammatory response and cytokine production [15,16,17]. In line with our findings, it has been suggested that a decrease in trace elements could be associated with poorer clinical outcomes and death in the setting of COVID-19 [17,29,30]. Low selenium plasma levels have been reported in critically ill patients, including in COVID-19 patients, and selenium supplementation has been proposed as an antioxidant adjunctive therapy in these patients [31]. Congruent with our results, others have reported that low selenium levels were associated with adverse outcomes in COVID-19 patients [32]. Copper plasma levels have been shown to be elevated in critically ill patients with systemic inflammation and in patients with COVID-19 [33]. In our study, copper levels were elevated in critically ill patients with COVID-19, but lower copper plasma levels were associated with prolonged time under MV.

Low trace element levels are a marker of disease severity and predictor of poor outcome, particularly in critically ill patients and in severe COVID-19 patients. However, it remains unclear whether a deficiency of these micronutrients is a risk factor for developing severe COVID-19 or whether it is simply a marker of disease severity [34]. A physiological explanation could be that these trace elements are excessively consumed in the context of severe COVID-19 and that the low levels at ICU admission simply reflect the severity of the disease. Although micronutrient measurement and substitution are not currently performed in the ICU, some centers have adopted this approach, although standardization of such practices remains elusive. Plasma trace elements levels should be interpreted with caution; because of shifts in trace elements during inflammation, low plasma levels may not be equivalent to deficiency [24,35].

An important question is whether supplementing these trace elements could influence the outcome of patients with severe infections and COVID-19. Studies on micronutrients substitution in critically ill COVID-19 patients are still scarce and have yielded conflicting results [36,37,38]. A recent RCT showed a decrease in mortality with zinc supplementation [39]. Other observational or smaller studies have shown an association with zinc supplementation and a reduction in the duration of MV [30,40].The supplementation of selenium and copper remains a matter of debate [32,41,42].Future studies should also take into account that the substitution of one of these micronutrients may affect the level of other elements; for example, zinc and copper are competitively absorbed in the gut [19].

This study has some limitations. First, the study design is monocentric. However, we do present a representative cohort of critically ill patients with COVID-19 and compared trace elements among three different cohorts of COVID-19 patients with different disease severity. Second, no data on nutritional intake were collected in this study, which could have affected the plasma levels of trace elements. Third, our study is limited in its ability to provide comprehensive information about the mechanisms underlying the observed changes in trace elements, as it only measured three specific trace elements at a specific time, which restricts the assessment of dynamic changes. Fourth, although we found an association between low plasma levels of trace element, disease severity and unfavorable outcome, reverse causation should be considered, as this study could not determine whether low micronutrient levels were a consequence or a contributing factor to the patients’ illness.

## 5. Conclusions

In this COVID-19 ICU cohort, lower plasma zinc levels tended to be associated with more septic shock and mortality, and lower levels of copper and selenium were associated with prolonged time under mechanical ventilation. These data suggest the need for further investigation through interventional trials in severe COVID-19 patients to assess the potential benefit of a systematic supplementation of zinc, copper and selenium.

## Figures and Tables

**Table 1:** Baseline characteristics of ICU COVID-19 patients.

	*n* = 118
Male gender, *n* (%)	91 (77.1%)
Age, median (IQR)	65 (57–73)
BMI, median (IQR)	28.1 (25.6–31.9)
Smoking status, *n* (%)	17 (14.4%)
Any comorbidity *, *n* (%)	94 (79.7%)
SAPS II on ICU admission, median (IQR)	52 (41–64)
CRP (mg/L) on ICU admission, median (IQR)	158 (102–208)
Leucocytes (g/L) on ICU admission, median (IQR)	7.9 (5.8–10.4)
Copper on ICU admission in µmol/L, median (IQR)	18.3 (16.2–20.5)
Zinc on ICU admission in µmol/L, median (IQR)	8.2 (6.9–9.7)
Selenium on ICU admission in µmol/L, median (IQR)	0.8 (0.7–1)
PaO_2_/FiO_2_ (kPa) on ICU admission, median (IQR)	18.6 (13.6–23.4)
Prone positioning, *n* (%)	89 (75.4%)
Number of Prone positioning session, median (IQR)	3 (2–4)
ECMO during ICU stay, *n* (%)	10 (8.5%)
Septic shock during ICU stay, *n* (%)	24 (20.3%)
Thrombosis event during ICU stay, *n* (%)	17 (14.4%)
Days under mechanical ventilation, median (IQR)	13 (9–17)
Length of stay in the ICU (days), median (IQR)	16 (11–22)
Length of stay in the hospital (days), median (IQR)	28 (19–40)
Mortality at day 28, *n* (%)	18 (15.3%)

Results are expressed as median (IQR) and *n* (%). * Comorbidities include any of the following: hypertension, diabetes, obesity, hypercholesterolemia, chronic obstructive pulmonary disease, chronic kidney disease, cardiomyopathy and cerebrovascular disease. Abbreviations: BMI, Body mass index; CRP, C-reactive protein; ECMO, extracorporeal membrane oxygenation; FiO_2_, inspired fraction of oxygen; PaO_2_, arterial partial pressure of oxygen; SAPS II, simplified acute physiology score II.

**Table 2:** Major outcomes according to level of trace elements (univariate analysis).

	Normal Zinc *n* = 59	Lower Zinc *n* = 59	*p*	Normal Copper *n* = 59	Lower Copper *n* = 59	*p*	Normal Selenium *n* = 59	Lower Selenium *n* = 59	*p*
Days under MV, median (IQR)	13 (8–17)	13 (10–19)	0.3	11 (8–15)	15 (11–21)	<0.01	12 (9–15)	13 (8–24)	0.09
Septic shock, *n* (%)	8 (13.6%)	16 (27.1%)	0.07	9 (15.3%)	15 (25.4%)	0.3	9 (15.3%)	15 (25.4%)	0.3
Mortality J28, *n* (%)	5 (8.5%)	13 (22%)	0.07	8 (13.6%)	10 (17%)	0.8	5 (8.5%)	13 (22%)	0.07

Results are expressed as median (IQR) and *n* (%). Two-sided Pearson’s chi-squared test for categorical variables, Mann–Whitney U test for continuous variables.

**Table 3:** Association between low levels of trace elements and outcomes (multivariate analysis).

	**Lower versus Normal Zinc, OR (CI 95%)**	***p***	**Lower versus Normal Copper, OR (CI 95%)**	***p***	**Lower versus Normal Selenium, OR (CI 95%)**	***p***
Septic shock *	2.6 (0.97–7)	0.06	1.8 (0.7–4.6)	0.2	1.9 (0.7–4.9)	0.2
Mortality *	1.9 (0.6–6.5)	0.3	2.3 (0.7–7.4)	0.2	2.6 (0.8–8.5)	0.1
	**Lower versus normal Zinc, β coefficient (CI 95%)**		**Lower versus normal Copper, β coefficient (CI 95%)**		**Lower versus normal Selenium, β coefficient (CI 95%)**	***p***
Days under MV *	2.3 (−0.9–5.5)	0.2	3.5 (0.4–6.6)	0.03	3.3 (0.2–6.3)	0.04

* estimates are adjusted to SAPSII score on ICU admission, gender and to the presence of any comorbidity. Results of the logistic regressions are expressed as odds ratio (OR) with 95% confidence intervals (CI95%). Results of the linear regressions are expressed as β coefficient and confidence intervals (CI 95%).

## Data Availability

After publication, the data will be made available to others on reasonable requests to the corresponding author. A proposal with detailed description of study objectives and statistical analysis plan will be needed for evaluation of the reasonability of requests. Additional materials might also be required during the process of evaluation. Deidentified participant data will be provided after approval from the corresponding author and University Hospitals of Geneva.

## Supplementary Materials

The following supporting information can be downloaded at: https://www.mdpi.com/article/10.3390/nu15153308/s1, Table S1: ICP-MS instrument operating conditions; Table S2: Trace elements acquisition parameters; Table S3: Analytical validation method; Table S4: Characteristics of patients by trace elements of ICU patients; Table S5: Correlation between trace elements levels in ICU patients.
