# Supplementary material for: Association of Trace Element Levels with Outcomes in Critically Ill COVID-19 Patients
Source: Nutrients. 2023 Jul 26;15(15):3308. doi: 10.3390/nu15153308 (PMC10421129; doi:10.3390/nu15153308)
Supplement: Supplementary file 1 [file nutrients-15-03308-s001.zip › nutrients-2515821-supplementary.pdf]

Supplementary Materials

Supplementary Table S1. ICP-MS instrument operating conditions.

**ICP-MS spectrometer**	**Agilent 7800 series**
Skimmer and sampler cones	Ni
Spray chamber	temperature stabilized (Peltier cooled)
Sample depth	8 mm
RF Power	1550 W
Plasma gas flow rate	15 L/min
Carrier gas flow rate	1.01-1.07 L/min
He flow rate (KED mode)	4.3 mL/min
Peristaltic pump tubing	inner Ø 1.02 mm, wall thickness 0.85 mm
Uptake time and nebulizer pump speed	50 s, 0.3 rps
Stabilization time and nebulizer pump speed	30 s, 0.1 rps
Oxyde (CeO/Ce) ratio	0.86-0.99 %
Doubly charged (Ce) ratio	1.08-1.25 %

Supplementary Table S2. Trace elements acquisition parameters.

Isotope	Integration time in KED mode (He)	Internal standard
^{63}Cu	0.5 s	Rhodium / Indium
^{66}Zn	0.5 s	Rhodium / Indium
^{78}Se / ^{82}Se	0.5 s	Rhodium / Indium

Supplementary Table S3. Analytical validation method.

CRM	Recipe ClinCkek-Control plasma level I and II			
Isotope	LOD (ng/ml)	LOQ (ng/ml)	Repeatability (%)	Reproducibility (%)
^{63}Cu	1.15	3.69	1.70	3.10
^{66}Zn	0.42	5.54	1.20	3.90
^{78}Se / ^{82}Se	0.61	1.68	1.40	2.50

Supplementary Table S4. Characteristics of patients by trace elements of ICU patients.

	Normal zinc N=59	Low zinc N=59	p	Normal copper N=59	Low copper N=59	p	Normal selenium N=59	Low selenium n=59	p
Sex male, n (%)	50 (84.8%)	41 (69.5%)	0.049	40 (67.8%)	51 (86.4%)	0.02	48 (81.4%)	43 (72.9%)	0.3
Age, median (IQR)	62 (54-69)	67 (59-76)	<0.01	62 (55-72)	66 (57-74)	0.2	59 (55-69)	68 (61-76)	<0.01
BMI, median (IQR)	29.1 (26-32.2)	27.5 (25.3-30.5)	0.1	29.1 (26-32.3)	27.8 (25.3-31.8)	0.2	29.1 (26-32.1)	27.8 (25.1-31.1)	0.08
Any comorbidity, n (%)	44 (74.6%)	50 (84.8%)	0.2	47 (79.7%)	47 (79.7%)	0.4	45 (76.3%)	49 (83.1%)	0.4
SAPSII on ICU admission, median (IQR)	50 (35-59)	58 (43-69	<0.01	53 (43-65)	50 (35-64)	0.3	51 (37-60)	57 (43-66)	0.2
CRP on ICU admission, median (IQR)	141 (83.9-180.3)	181 (123-236)	<0.01	165.7 (112-209.1)	138 (95.3-283.7)	0.3	128 (81.9-168.1)	184.6 (123.2-239)	<0.01
Leucocytes count on ICU admission, median (IQR)	6.9 (5.5-10)	8.7 (6.2-10.8)	0.1	8 (6.2-10.7)	7.6 (5.2-10.4)	0.4	6.7 (5.5-9.7)	9 (5.9-11.1)	0.03
Copper on ICU admission in umol/l, median (IQR)	19.1 (16.4-21.6)	17.8 (15.9-19.6)	0.03	20.5 (19.3-22.6)	16.2 (15-17.3)	<0.01	19.4 (17.4-22.2)	17.2 (15.3-18.8)	<0.01
Zinc on ICU admission in umol/l, median (IQR)	9.7 (8.6-11.7)	6.9 (6.1-7.6)	<0.01	8.5 (7.3-10.2)	7.7 (6.5-9)	0.02	8.5 (7.4-10.4)	7.5 (6.3-9)	<0.01
Selenium on ICU admission in umol/l, median (IQR)	0.9 (0.8-1.1)	0.8 (0.7-1)	<0.01	0.9 (0.8-1.1)	0.8 (0.7-0.9)	<0.01	1 (0.9-1.1)	0.7 (0.7-0.8)	<0.01
PaO2/FiO2 on ICU admission, median (IQR)	19.8 (13.7-26.1)	17.7 (12.4-20.7)	0.5	18.6 (13.6-25)	18.9 (13.5-21.4)	0.9	18.2 (13.7-25.7)	19 (13-21)	0.06
ECMO during ICU stay, n (%)	3 (5%)	7 (11.9%)	0.2	5 (8.5%)	5 (8.5%)	1	5 (8.5%)	5 (8.5%)	1
Septic shock during ICU stay, n (%)	8 (13.6%)	16 (27.1%)	0.07	9 (15.3%)	15 (25.4%)	0.3	9 (15.3%)	15 (25.4%)	0.2
Time under mechanical ventilation, median (IQR)	13 (8-17)	13 (10-19)	0.3	11 (8-15)	15 (11-21)	<0.01	12 (9-15)	13 (8-24)	0.09
ICU LOS, median (IQR)	15 (10-21)	16 (11-25)	0.5	13 (10-20)	17 (13-24)	<0.01	15 (11-19)	16 (10-26)	0.3
Mortality at day 28, n (%)	5 (8.5%)	13 (22%)	0.07	8 (13.6%)	10 (17%)	0.8	5 (8.5%)	13 (22%)	0.04

Supplementary Table S5. Correlation between trace elements levels in ICU patients.

	Zinc	Copper	Selenium
Zinc	**1**		**0.3 p<0.01**
Copper	**0.3 p<0.01**	**1**	
Selenium		**0.5 p<0.01**	**1**

A Pearson's product-moment correlation was run to assess the relationship between copper, zinc and selenium levels in the 118 ICU patients. There was a moderate correlation between zinc and copper levels ($r=0.3$, $p<0.01$), between copper and selenium levels ($r=0.5$, $p<0.01$) and between selenium and zinc ($r=0.3$, $p<0.01$).
